# PET‐based radiomics signature can predict durable responses to CAR T‐cell therapy in patients with large B‐cell lymphoma

**DOI:** 10.1002/jha2.757

**Published:** 2023-09-11

**Authors:** Marta Ligero, Marc Simó, Cecilia Carpio, Gloria Iacoboni, Maria Balaguer‐Montero, Victor Navarro, Mario Andres Sánchez‐Salinas, Sabela Bobillo, Ana Marín‐Niebla, Josu Iraola‐Truchuelo, Pau Abrisqueta, Roser Sala‐Llonch, Francesc Bosch, Raquel Perez‐Lopez, Pere Barba

**Affiliations:** ^1^ Radiomics Group Vall d'Hebron Institute of Oncology (VHIO) Vall d'Hebron Barcelona Hospital Campus (VHUH) Barcelona Spain; ^2^ Nuclear Medicine Department Vall d'Hebron University Hospital, Autonomous University of Barcelona Barcelona Spain; ^3^ Department of Hematology Experimental Hematology, Vall d’Hebron Institute of Oncology (VHIO), Vall d’Hebron University Hospital Barcelona Barcelona Spain; ^4^ Oncology Data Science (ODysSey) Group Vall d'Hebron Institute of Oncology (VHIO) Barcelona Spain; ^5^ Faculty of Medicine Department of Biomedicine Institute of Neurosciences, Institut d'Investigacions Biomèdiques August Pi i Sunyer (IDIBAPS) University of Barcelona Barcelona Spain; ^6^ Centro de Investigación Biomédica en Red de Bioingeniería Biomateriales y Nanomedicina (CIBER‐BBN) Barcelona Spain

**Keywords:** CAR T cells, DLBCL, non‐Hodgkin lymphoma, PET, progression‐free survival, radiomics

## Abstract

Chimeric antigen receptor (CAR) T‐cell therapy is a promising treatment option for relapsed or refractory (R/R) large B‐cell lymphoma (LBCL). However, only a subset of patients will present long‐term benefit. In this study, we explored the potential of PET‐based radiomics to predict treatment outcomes with the aim of improving patient selection for CAR T‐cell therapy. We conducted a single‐center study including 93 consecutive R/R LBCL patients who received a CAR T‐cell infusion from 2018 to 2021, split in training set (73 patients) and test set (20 patients). Radiomics features were extracted from baseline PET scans and clinical benefit was defined based on median progression‐free survival (PFS). Cox regression models including the radiomics signature, conventional PET biomarkers and clinical variables were performed for most relevant outcomes. A radiomics signature including 4 PET‐based parameters achieved an AUC = 0.73 for predicting clinical benefit in the test set, outperforming the predictive value of conventional PET biomarkers (total metabolic tumor volume [TMTV]: AUC = 0.66 and maximum standardized uptake value [SUV_max_]: AUC = 0.59). A high radiomics score was also associated with longer PFS and OS in the multivariable analysis. In conclusion, the PET‐based radiomics signature predicted efficacy of CAR T‐cell therapy and outperformed conventional PET biomarkers in our cohort of LBCL patients.

## Graphical abstract

In patients with large B‐cell lymphoma treated with CAR T‐cells,radiomics quantification of the PET component allows for better prediction of short term recurrence(3‐moths PFS)than conventional SUVmax and Total Metabolic Tumor Volume(TMTV)

The combination of Radiomics signature with clinical data allows for better patient stratification

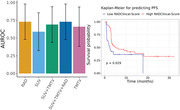

## 1 INTRODUCTION

Diffuse large B‐cell lymphoma (DLBCL) is the most common type of non‐Hodgkin lymphoma [1]. Despite durable responses to first‐line immunochemotherapy in approximately 60% of cases [2, 3], less than half of relapsed/refractory (R/R) patients are salvaged with intensive chemotherapy followed by autologous stem cell transplant consolidation [4]. Chimeric antigen receptor (CAR) T‐cell therapy is the established third‐line treatment for this disease [5, 6] and has shown significantly improved results in comparison with chemotherapy for early relapsed and refractory patients in the second‐line setting [7, 8]. However, not all patients experience the same clinical benefit with this cellular therapy, raising the need to find predictive biomarkers to aid patient selection, leading toward a better efficacy and safety profile.

Quantitative imaging parameters from ^18^F‐fluorodeoxyglucose (FDG) positron emission tomography (PET) such as total metabolic tumor volume (TMTV) and the maximum standardized uptake value (SUV_max_) have been associated with outcomes in patients with DLBCL [9, 10, 11, 12]. However, these standard quantitative PET characteristics are limited to tumor burden and maximum metabolic consumption, but do not provide details about the phenotype or spatial distribution of the malignancy. Also, the reported results of these parameters are heterogeneous, and their routine clinical use is still limited [9, 11, 13, 14, 15].

PET‐based radiomics provides quantitative imaging data regarding tumor shape, heterogeneity, and metabolic consumption distribution. These parameters can provide insight into the tumor biology, including meaningful information about the cancer cell metabolism and tumor microenvironment for further identification of responsive phenotypes. Some studies have defined PET‐based radiomic features associated with a favorable response to standard chemotherapy in patients with DLBCL [16, 17]. However, scarce data is available in the specific setting of CAR T‐cell therapy [18].

In this study, we analyzed FDG‐PET‐based radiomic phenotypes in R/R DLBCL patients treated with CD19‐targeted CAR T‐cell therapy, aiming to identify those patients who would benefit most from treatment. Furthermore, we looked at the added value of textural features to standard prognostic factors for efficacy and safety after therapy.

## 2 METHODS

### 2.1 Study population

The study included all consecutive patients with R/R diffuse large B‐cell lymphoma (DLBCL), de novo or transformed from an indolent lymphoma, who received a single infusion of CD19‐targeted second‐generation CAR T cells from July 2018 to November 2021 at Vall d'Hebron University Hospital and had at least 1 disease assessment after infusion. For both cytokine release syndrome (CRS) and immune effector cell‐associated neurotoxicity syndrome (ICANS), grading was carried out according to the American Society for Transplantation and Cellular Therapy (ASTCT) criteria and management followed local guidelines [19, 20]. All patients included in the study provided informed consent. The study was approved by the ethical committee of the Vall d'Hebron University Hospital (PR[AG]404/2020) and conducted in accordance with the Declaration of Helsinki.

### 2.2 Endpoints and definitions

Clinical data were collected from the electronic medical records. These included age, sex, disease stage before CAR T‐cell therapy, previous lines of treatment, previous stem cell transplant (SCT), Eastern Cooperative Oncology Group (ECOG) status, lactate dehydrogenase (LDH), tumor histology (transformed from indolent lymphoma or DLBCL de novo high‐grade B‐cell lymphoma double hit/triple hit [DH/TH]) and cell of origin (germinal center B‐cell [GCB] or non‐GCB). The primary endpoint was progression‐free survival (PFS), defined as time between CAR T‐cell infusion and disease progression or death, whichever occurred first. Secondary endpoints included overall survival (OS), defined as the time between CAR T‐cell infusion and death, best response achieved after infusion and clinically significant adverse events, defined as grade ≥2 CRS and/or ICANS in this study.

### 2.3 Disease assessment and evaluation

All patients underwent a PET‐CT scan at our institution after the last bridging regimen, within 7 days of starting lymphodepleting chemotherapy. Disease evaluation after CAR T‐cell therapy was scheduled at 1, 3, 6, 12, 18, and 24 months after infusion. The imaging reports were based on the Lugano recommendations for response assessment and graded according to the 5‐point Deauville score [21]. Patients achieving a complete metabolic response (CMR, Deauville scores 1–3) or partial metabolic response (PMR) were considered responders to CAR T‐cell therapy.

### 2.4 PET/CT exam and feature acquisition

The PET‐CT exam required a minimum of 6‐hour fast prior to the intravenous administration of 3.7 MBq/kg (222–370 MBq) of ^18^fluorodeoxyglucose (18F‐FDG). Glucose values below 140 mg/dL were required in all cases prior to administration of the radiopharmaceutical. Before scanning, the patients were at rest for a minimum of 60 min. Images were obtained using a Siemens Biograph mCT, which combines a spiral CT of 64 slices (210 keV, 120 mAs, care dose) with a dedicated PET, from the skull to the upper third of both femurs. The images generated were evaluated by a nuclear medicine physician in a syngo.via Siemens Healthcare workstation [9].

Standard Uptake Value (SUV) was computed voxel‐wise from ^18^F‐FDG PET/CT images normalized to patient's weight and injected dose [22]. Lesions were delineated in the PET scans by an experienced nuclear medicine physician using a custom workflow from MIM Encore™ software (MIM Software Inc., Cleveland, OH, USA), which selected all metabolic activity above the 41% maximum SUV threshold. Images and segmentations were reviewed by a nuclear medicine specialist. Before feature extraction, images were resampled to 2 × 2 × 2 mm^3^ using spline interpolation and discretized to a fixed bin number of 25 [23]. From the lesion with the largest volume, 105 radiomics features were extracted. These features correspond to quantitative characteristics regarding first order SUV distribution and metabolic consumption heterogeneity in the tumor measured by Gray‐Level Co‐occurrence Matrix [GLCM], Gray‐Level Dependence Matrix [GLDM], Gray‐Level Run Length Matrix [GLRLM], and Gray‐Level Size Zone Matrix [GLSZM]). All features were computed as described by van Griethuysen et al. [24]. Conventional PET features (SUV_max_ and TMTV) were also computed from the whole metabolic disease. Image preprocessing and feature extraction were computed using Pyradiomics v.3.0.1 [24] package and Python v.3.8.8.

### 2.5 Statistical analysis

**FIGURE 1:**
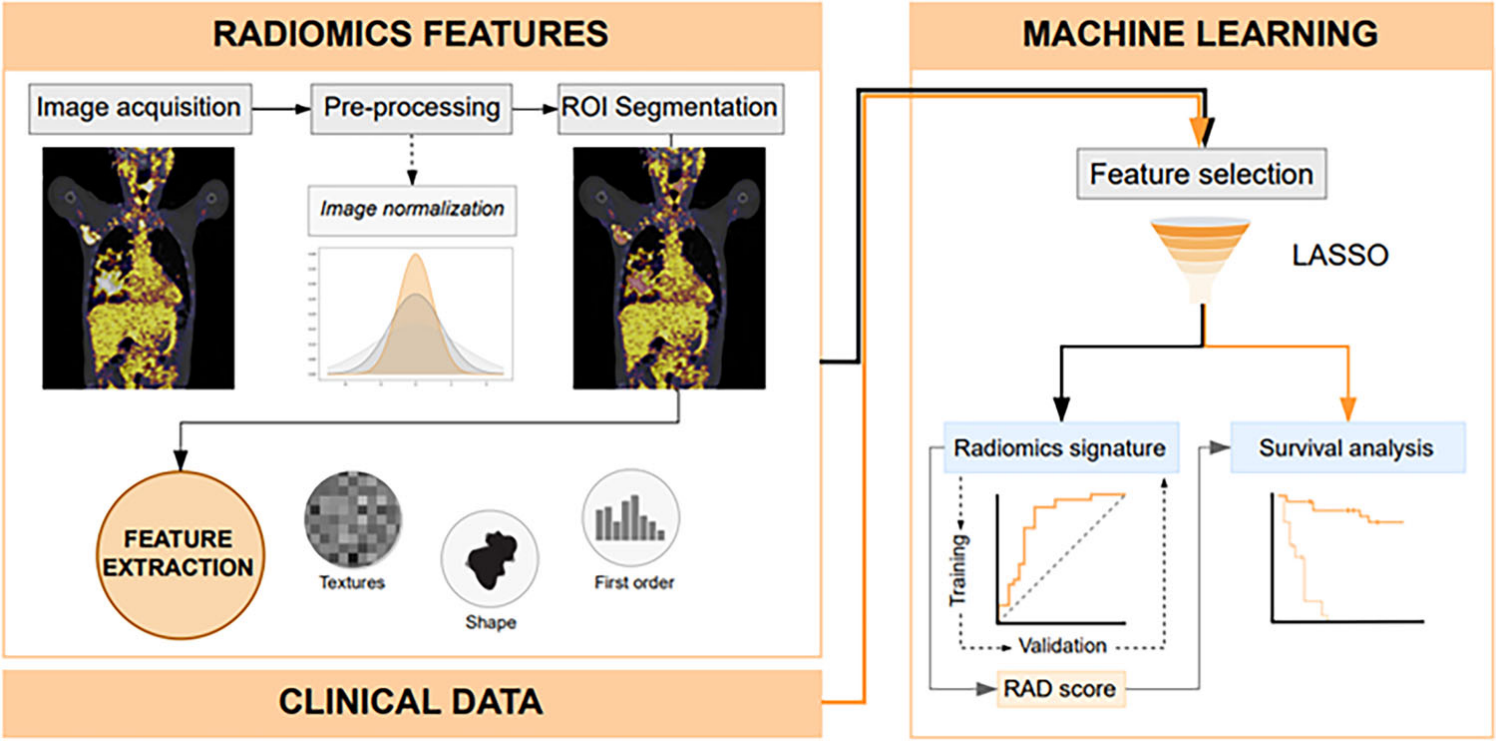
Radiomics analysis workflow.

Data was split into training and test sets (80% and 20%, respectively). The training set was used to build the model and the test set to validate its performance. Population characteristics of both sets were balanced according to baseline international prognostic index (IPI) score, costimulatory domain and treatment response. In the training cohort, the least absolute shrinkage and selection operator (LASSO)‐regression were used for radiomic feature selection. LASSO regularization parameter (*λ*) was optimized by maximizing the area under the curve (AUC) from cross‐validation. Logistic regression including the selected features was trained and tested for classifying 3‐month PFS (median PFS of this cohort). The performance of conventional PET features and the radiomic score were compared using the AUC from the receiver operating characteristic (ROC) curve. The 95% confidence intervals and the *p* value were assessed with the DeLong method and the Mann–Whitney *U* test, respectively. Stratified repeated fivefold cross‐validation was implemented to explore the model generalizability for different data splits. Finally, univariate and multivariate Cox proportional hazard regression analysis were performed with clinical and PET‐based data (including the radiomics signature) to investigate the impact of these factors on continuous PFS and OS. Kaplan–Meier curves were reported for the multivariate Cox prediction after discretization by Youden's threshold optimization (Figure 1). The potential impact of radiomic features on safety outcomes (grade ≥2 CRS and/or ICANS) were evaluated using the Mann–Whitney *U* test.

## 3 RESULTS

### 3.1 Patient characteristics

**FIGURE 2:**
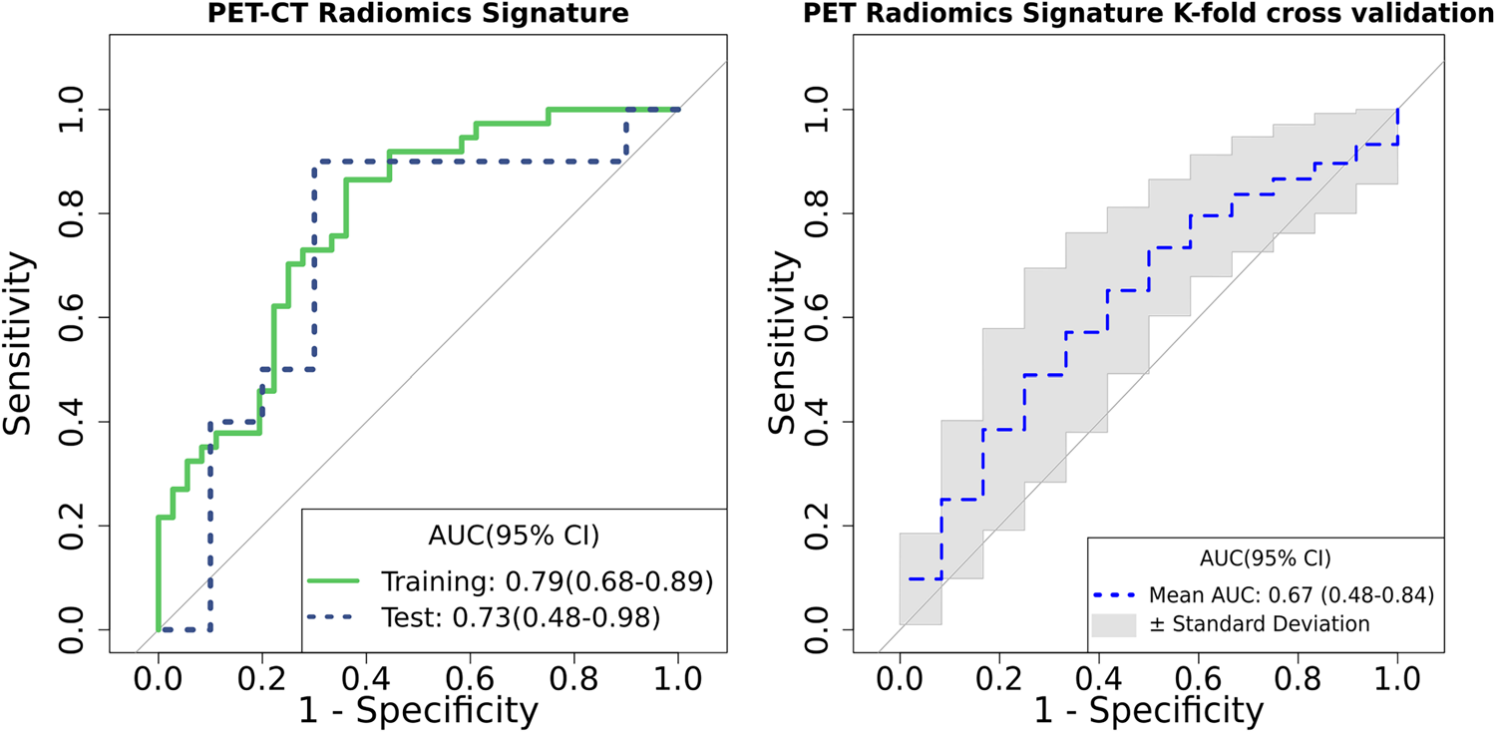
ROC curves for 3‐month progression‐free survival (PFS) prediction of the radiomics signature in the training and test sets (A) and fivefold cross‐validation analysis (B).

Of 101 consecutive patients with R/R DLBCL treated with CD19‐targeted CAR T cells during the study period, 7 were excluded because pretreatment PET scans were not available or disease was not measurable, and 1 patient died prior to the first response evaluation (Figure 2). Finally, 93 patients were included in the study.

Patients’ characteristics are summarized in Table 1. The median age was 59 years (interquartile range [IQR] 50–68) and 68% were male. Median number of prior lines was 2 (IQR 2–3), 73% had an advanced stage of disease, and 33% had undergone a previous autologous stem cell transplant (ASCT). Regarding the construct, 33% received axicabtagene ciloleucel and 67% received a CD19‐targeted construct with 4‐1BB costimulatory domain, including tisagenlecleucel and an investigational product. Median values for SUV_max_ and TMTV were 16 [IQR 21–22] and 177 cm^3^ [45–549], respectively.

**TABLE 1:** Baseline patient characteristics.

Characteristics	*N* = 93	Training *N* = 73	Test *N* = 20	p*
**Age, years, median (IQR)**	59 (50–68)	57 (47–67)	62 (56–66)	0.115
Age, ≥65 years, *n* (%)	30 (32)			
**Sex, male, *n* (%)**	63 (68)	48 (66)	25 (34)	0.590
**Disease stage before CAR T‐cell therapy**				
Stages I–II, *n* (%)	25 (27)	21(29)	4 (20)	
Stages III–IV, *n* (%)	68 (73)	52 (71)	16 (80)	0.573
**Previous lines of treatment**				
>2, *n* (%)	31 (33)	23 (32)	8 (40)	0.593
Previous SCT, *n* (%)	30 (32)	21 (29)	9 (45)	0.186
ECOG, ≥1, *n* (%)	61 (66)	46 (63)	15 (75)	0.428
**Primary refractory**, *n* (%)	55 (59)	41 (56)	14 (70)	0.312
**Median LDH/ULN** (IQR), U/L	1.39 (0.93–2.28)	1.39 (0.94–2.24)	1.04 (0.93–2.39)	0.728
**LDH > 2 × ULN**, *n* (%)	32 (34)	26 (36)	6 (30)	0.792
**IPI prognostic score**, *n* (%)				
0–2	49 (52)	37 (51)	12 (60)	
3–5	44 (47)	36 (49)	8 (40)	0.614
**Bulky disease (>7 cm)**, *n* (%)	30 (32)	23 (32)	9 (45)	0.294
**Histology**, *n* (%)				
Transformed from indolent lymphoma	20 (21)	15 (21)	5 (25)	0.760
DLBCL de novo	61 (66)	48 (65)	(66)	1
High‐grade B‐cell lymphoma (DH/TH)	12 (13)	10 (14)	2 (10)	1
**Cell of origin**, *n* (%)				
GCB	60 (64)	49 (67)	11 (55)	
Non‐GCB	25 (27)	19 (26)	6 (30)	
Not available	8 (9)	5 (6)	3 (15)	0.416
**Costimulatory domain**, *n* (%)				
41BB	62 (67)	49 (67)	13 (65)	
CD28	31 (33)	24 (33)	7 (35)	1

^*^ *p* Values were obtained from ANOVA test for continuous variables and Fisher's exact test for categorical variables.

### 3.2 Efficacy and safety data

Best response after CAR T‐cell therapy was CMR in 37 (40%) patients, PMR in 33 (35%) (overall response rate 75%) and PMD in 23 (25%). With a median follow‐up of 17.6 months (95% CI 12–24.3), median PFS and OS of the whole cohort were 3.9 months (95% CI 3.03–14.1) and 15.4 months (95% 10.8–NR), respectively.

Regarding toxicity, any grade of CRS and ICANS occurred in 68 (73%) and 33 (35%) patients, respectively. Grade ≥2 CRS and ICANS developed in 29 (31%) and 25 (27%) patients, respectively.

### 3.3 Radiomics signature development

Seventy‐three and 20 patients were randomly selected to build (training set) and validate (test set) the model. Baseline characteristics were similar between the training and test sets (Table 1). Among the 105 computed radiomics features, four of them were identified as those with the highest association with PFS using LASSO with the lambda of λ = 0.102 as described in the methods. The 4 selected features included maximum intensity, skewness, major axis length, and large dependence low gray‐level emphasis (LDLGLE) from the texture matrix GLDM. The radiomics scored can be obtained by using Equation (1)

(1) $$RadScore\;=\;\frac{1}{1+{\;\exp}^{-y}}$$

$$\begin{array}{c} y\;=\;-0.042+\;-0.580\times Shape\;Major\;Axis\;Length\;+\\ -0.331\times \;First\;order\;Maximum\;+\\ -0.646\times First\;order\;Skewness\;\times \\ -0.565\times GLDM\;LDLGLE. \end{array}$$

The PET‐based radiomics signature points out that having larger lesions (measured by major axis length) negatively contribute to have a worse prognosis. Similarly, lesions with high SUV_max_ (maximum intensity) or negatively skewed distribution (skewness), which means more density of voxels in with high SUV values are more likely to not benefit more from CAR T‐cell therapy (Table 3). Additionally, the model also includes GLDM large dependence low gray‐level emphasis, which indicates that lesions with more homogeneity in lower SUV_max_ values will have less risk of progress. The radiomics score reached an AUC of 0.79 [95% CI 0.68–0.89] and 0.73 [95% CI 0.48–0.98] in the training and test sets, respectively, for prediction of 3‐month PFS. Moreover, the radiomics signature showed a stable classification performance with an AUC of 0.67 [95% CI 0.48–0.84] in the cross‐validation analysis (Figure 2). Accuracy, sensitivity, and specificity for the optimal cut‐off of 0.43 are described in Table 2.

**TABLE 2:** Radiomics versus conventional PET performance using AUC, accuracy (ACC), sensitivity (SE), specificity (SP), positive predictive value (PPV), and negative predictive value (NPV).

	Training							Test						
	AUC [95% CI]	*p* Value	ACC (%)	SE (%)	SP (%)	NPV (%)	PPV (%)	AUC [95% CI]	*p* Value	ACC (%)	SE (%)	SP (%)	NPV (%)	PPV (%)
**RAD score**	0.79 [0.68–0.89]	**<0.001**	75	87	64	82	71	0.73 [0.48–0.98]	0.045	65	40	90	80	60
**SUV_max_**	0.66 [0.53–0.79]	0.992	67	59	75	82	71	0.59 [0.32–0.86]	0.759	55	70	40	57	54
**TMTV**	0.66 [0.53–0.78]	0.99	66	81	50	72	63	0.66 [0.38–0.94]	0.891	60	80	40	67	57

Abbreviations: RAD, radiomics score; SUV_max_, maximum standard uptake value; TMTB, total metabolic tumor burden.

In bold *p*‐value below 0.05 (i.e., statistically significant).

### 3.4 Integration of radiomics and conventional PET features to predict CAR T‐cell efficacy

**FIGURE 3:**
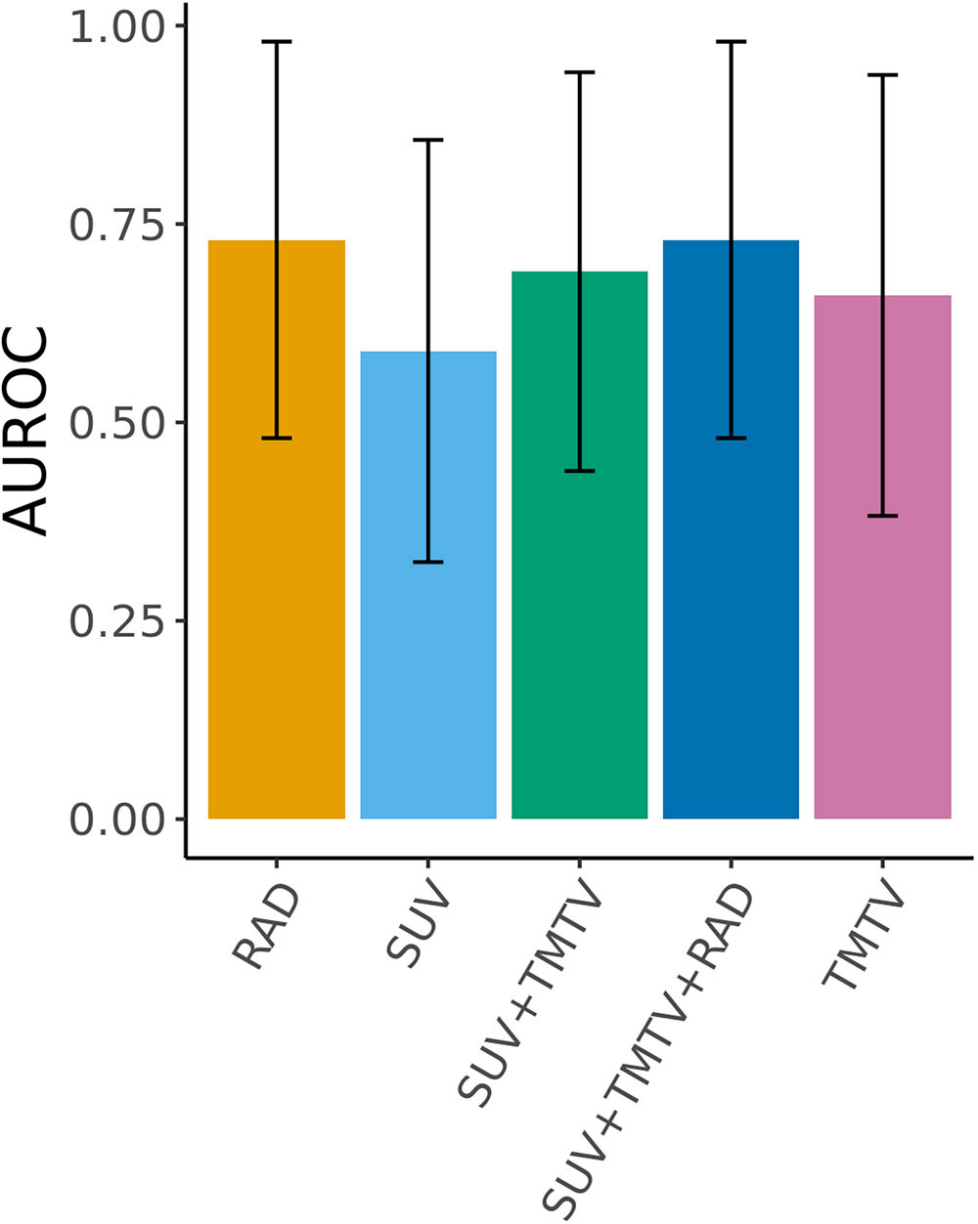
Area under the reciever operating characteristic (AUROC) curve for 3‐month progression‐free survival (PFS) prediction of conventional PET biomarkers (SUV_max_ and TMTV) and radiomics score individually or incombination. RAD, radiomics score; SUV, standard uptake value; TMTB, total metabolic tumor burden.

Thereafter, we investigated the performance of conventional PET features to predict PFS in our population. PET established biomarkers including baseline SUV_max_ of all lesions (AUC 0.59 [95% CI 0.32–0.86]) and TMTV (AUC 0.66 [95% CI 0.38–0.94]), individually or in combination (AUC 0.69 [95% CI 0.44–0.94]), showed a lower predictive capacity than the radiomics score for 3‐month PFS. Furthermore, the addition of preestablished PET parameters to the radiomics score did not increase the predictive capacity of the latter in the training nor test sets (Figure 3).

### 3.5 Impact of imaging and clinical factors on efficacy

**FIGURE 4:**
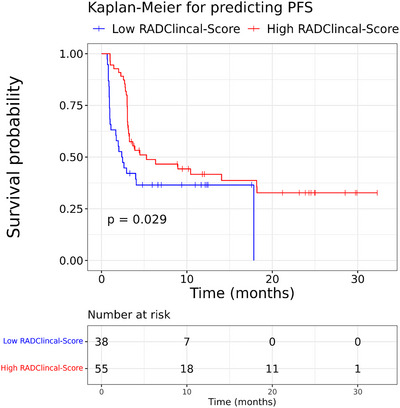
Kaplan–Meier curves for the multivariate analysis predicted progression‐free survival (PFS) values.

In the univariate analysis, a low radiomics score (HR 2.97 [95% CI 1.74–5.1], *p* < 0.001), high baseline TMTV (HR 2.01 [95% CI 1.27–3.17], *p* = 0.003) and SUV_max_ (HR 1.03 [95% CI 1.00–1.05], *p* = 0.022), as well as ECOG ≥1 at lymphodepletion (HR 1.91 [95% CI 1.06–3.44], *p* = 0.031), a higher number of previous lines of therapies (HR 1.71 [95% CI 1.29–2.27], *p* < 0.001) and 4‐1BB as costimulatory domain (HR 4.17 [95% CI 2.04–8.53], *p* < 0.001) were associated with a shorter PFS. The low radiomics score and costimulatory domain 4‐1BB retained significance in the multivariate LASSO‐Cox regression analysis for PFS (Figure 4, Table 3).

**TABLE 3:** Univariate and multivariate Cox for progression‐free survival (PFS) and overall survival (OS).

	Univariate and multivariate analysis for PFS				Univariate and multivariate analysis for OS			
	Univariate analysis		Multivariate analysis		Univariate analysis		Multivariate analysis	
	HR [95% CI]	*p*	HR [95% CI]	*p*	HR [95% CI]	*p*	HR [95% CI]	*p*
Age	1.01 [0.99–1.04]	0.223			1.01 [0.99–1.04]	0.299		
ECOG ≥1	1.91 [1.06–3.44]	**0.031**			2.39 [1.14–5.03]	0.021	1.83 [0.84–3.97]	0.127
Number of previous lines of treatment	1.71 [1.29–2.27]	**<0.001**			1.89 [1.36–2.63]	<0.001	1.85 [1.27–2.69]	0.001
Costimulatory domain 4‐1BB	4.17 [2.04–8.53]	**<0.001**	3.90 [1.9–8.00]	**<0.001**	2.81 [1.24–6.35]	0.013	1.89 [0.81–4.4]	0.138
TMTV (dL)	2.01 [1.27–3.17]	**0.003**			2.33 [1.41–3.86]	0.001	1.44 [0.79–2.61]	0.236
SUV_max_	1.03 [1.01–1.05]	**0.022**			1.03 [1–1.06]	0.024		
Low RAD score	2.97 [1.47–5.10]	**<0.001**	2.72 [1.59–4.67]	**<0.001**	2.83 [1.52–5.27]	<0.001	2.33 [1.2–4.51]	<0.001

*Note*: All parameters were assessed from the pretreatment PET imaging.

In bold *p*‐value below 0.05 (i.e., statistically significant).

Regarding OS, a low radiomics score (HR 2.83 [95% CI 1.52–5.27], *p* < 0.001), high baseline TMTV (HR 2.33 [95% CI 1.35‐ 3.86], *p* = 0.001), and SUV_max_ (HR 1.03 [95% CI 1.01–1.06], *p* = 0.024), as well as for ECOG ≥1 (HR 2.39 [95% CI 1.14–5.03], *p* = 0.021), higher number of previous lines of treatment (HR 1.89 [95% CI 1.36–2.63], *p* < 0.001) and 4‐1BB as costimulatory domain (HR 2.81 [95% CI 1.24–6.35], *p* = 0.013) were associated with a lower OS in the univariate analysis. In the multivariate LASSO‐Cox model for OS, the radiomics score, ECOG ≥1, 4‐1BB as costimulatory domain, baseline TMTV, and number of previous lines of treatment retained a significant impact (Table 3).

### 3.6 Impact of radiomics and conventional PET‐based features on safety

Finally, we explored the association between the radiomics score and conventional PET‐based features on significant adverse events after CAR T‐cell therapy. Neither the radiomics score TMTV nor SUV_max_ was associated with a higher incidence of grade ≥2 CRS or ICANS in our cohort (*p* > 0.05). Similarly, we could not identify risk factors for grade ≥2 CRS and/or ICANS among the clinical variables including age, ECOG ≥1, number of previous lines of treatment, and costimulatory domain (*p* > 0.05).

## 4 DISCUSSION

The advent of CAR T‐cell therapy has significantly improved the prognosis of patients with R/R DLBCL. However, only a subset of patients achieves durable responses after therapy. The identification of predictive biomarkers to aid patient selection and prognostic stratification is an unmet need in the field. In this study, we developed a PET‐based radiomics signature capable of predicting clinical benefit after CAR T‐cell therapy with an accuracy of 75%. This radiomics signature combined metabolic consumption (SUV), tumor size, and metabolic activity distribution, indicating that patients with smaller lesions and lower SUV_max_ or negatively skewed distribution are more likely to benefit from CAR T‐cell therapy. The radiomics signature outperformed other established PET parameters, indicating that a more comprehensive evaluation of PET images including radiomic features could, more accuratelycapture, which DLBCL patients are more likely to respond to this novel cellular therapy.

Conventional PET‐based biomarkers such as TMTV and SUV_max_ have been analyzed as prognostic biomarkers in lymphoma patients treated with CAR T cells [9, 10, 11], showing that higher baseline values are associated with a lower probability of response and long‐term survival. Moreover, PET‐based radiomic signatures have been associated with response to treatment in DLBCL patients treated with conventional chemotherapy, showing a better performance than standard clinical scoring systems, such as the IPI score [16]. However, few studies have implemented PET‐based radiomics for modeling tumor response to CAR T‐cell therapy. Zhou et al. evaluated the prognostic value of different PET‐based radiomic features and explored the association between baseline radiomics and cytokine release syndrome in a small cohort of lymphoma patients. Similar to our results, Zhou et al. found that some textural features had a better performance than conventional PET parameters. Reinert et al. identified temporal associations between radiomic textural features and serologic markers of response such as C‐reactive protein, LDH and leukocyte count. Noteworthy, these studies were performed in limited cohorts (less than 30 patients each) and only carried out univariate analysis to associate PET‐based radiomic features to outcome. In our study, a multivariate analysis combining different tumor characteristics was performed to define responsive phenotypes. Furthermore, the availability of a larger cohort of patients treated with CAR T cells allowed us to test our model using internal validation.

In our study, tumor heterogeneity by radiomics evaluation was associated with a lower probability of response after CAR T‐cell therapy. This could be explained by a diverse composition of the tumor cells and microenvironment and, potentially, heterogeneous expression of the target antigen, which have been described as predictive biomarkers of response in the setting of adoptive T‐cell therapies [25, 26].

Several limitations should be taken into account in this study. Despite being one of the largest studies on radiomics in CAR T‐cell patients, our results are limited by the relatively small sample size of the validation cohort. Nevertheless, the results are encouraging to explore further in larger, more heterogeneous datasets, allowing an improvement of the model and additional validation. Of note, our pipeline incorporates early preprocessing steps including SUV standardization, making our model easier to be computed from images acquired with different scanners and facilitating its implementation in clinical practice. Furthermore, we have provided the preprocessing methods as well as the equation to obtain the final score. This will facilitate the validation in of our signature in other external cohorts without needing the data access. Moreover, further analysis of these biomarkers, integrating clinical, imaging and molecular data, could lend insight into the biological mechanisms that lead to higher response rates in these patients. Thus, the indication of CAR T‐cell therapy should not be made based on a single variable but integrating radiomics with other prognostic factors.

In conclusion, in this study, we trained and validated a proof‐of‐concept PET‐based radiomics signature to predict response to CAR T cells. This model outperformed conventional PET parameters and showed an independent predictive value after adjusting for well‐known clinical factors.

## AUTHOR CONTRIBUTIONS

Study design and development: RPL, PB, MS, GI, ML, MB. Data acquisition, management, and retrieval: MS, MB, CC, PB, GI, VN, MSz, SB, AM‐N, JI, PA. Image manual segmentation: MS, MB. Data preprocessing, analysis, and inference: ML, MB, VN. Interpretation and validation of results: ML, RPL, PB, GI, RSL, VN. All of the authors reviewed and approved the manuscript for its publication and certify the integrity of the presented work.

## CONFLICT OF INTEREST STATEMENT

PB receives honoraria from Allogene, Amgen, BMS, Gilead, Miltenyi Biomedicine, Pierre Fabre, Nektar, Novartis, and Pfizer. GI receives consultancy and honoraria from Novartis, Roche, Kite/Gilead, Bristol‐Myers Squibb, Abbvie, Janssen, Sandoz, Miltenyi, and AstraZeneca. SB receives honoraria from AstraZeneca, ROCHE, Janssen, and Abbvie. This work was partially presented at the 64th Annual Meeting of the American Society of Hematology, 2022.

## FUNDING INFORMATION

This research received no external funding. RPL is supported by LaCaixa Foundation, a CRIS Foundation Talent Award (TALENT19‐05), the FERO Foundation, the Instituto de Salud Carlos III‐Investigacion en Salud (PI18/01395 and PI21/01019), and the Prostate Cancer Foundation (18YOUN19). ML is supported by the PERIS PIF‐Salut Grant (SLT017/20/000080).

## ETHICS STATEMENT

The study was approved by the ethical committee of the Vall d'Hebron University Hospital (PR[AG]404/2020) and conducted in accordance with the Declaration of Helsinki.

## PATIENT CONSENT STATEMENT

All patients included in the study provided informed consent

## Data Availability

The raw data of imaging scans analyzed in this study are not publicly available due to their containing information, as this would compromise the privacy of research participants. Any queries for data access used in this study should be directed to the corresponding author.
